# Cost–consequence analysis of fluticasone furoate/vilanterol for asthma management in Spain: an analysis based on the Salford Lung Study in asthma

**DOI:** 10.1007/s10198-019-01101-x

**Published:** 2019-09-23

**Authors:** Laura Amanda Vallejo-Aparicio, Jesús Molina, Iñigo Ojanguren, Ana Viejo Casas, Alicia Huerta, Henrik Svedsater

**Affiliations:** 1 Market Access, GSK (GlaxoSmithKline), Madrid, Spain; 2 Centro de Salud Francia, Fuenlabrada, Madrid Spain; 3 Servicio de Neumología, Hospital Universitario Vall d’Hebron, Barcelona, Spain; 4 Centro de Investigación en Red de Enfermedades Respiratorias (CIBERES), Instituto de Salud Carlos III (ISCIII), Barcelona, Spain; 5 Centro de Salud Pisueña Cayon, Cantabria, Spain; 6 Value Evidence and Outcomes, GSK, Brentford, UK

**Keywords:** Asthma, Fluticasone furoate/vilanterol, Salford Lung Study, Costs, Corticosteroids, Control, I18, H61

## Abstract

**Objectives:**

The Salford Lung Study in asthma (SLS asthma) is a 12-month, open-label randomised clinical trial comparing clinical effectiveness of initiating once-daily inhaled combination of fluticasone furoate/vilanterol (FF/VI) 184/22 mcg or 92/22 mcg, with continuing optimized usual care (UC) with inhaled corticosteroids (ICS) alone, or in combination with a long-acting β2-agonist (ICS/LABA), in asthmatic patients followed in primary care in the UK. The objective of the analysis is to estimate the economic impact of these results when applied in Spain.

**Methods:**

A 1-year cost–consequence model was populated with SLS asthma, adopting the Spanish National Health System (NHS) perspective. 775,900 of diagnosed asthmatic patients ≥ 18 years old currently managed with UC in Spain were included in the analysis. Effectiveness data included the percentage of patients per Asthma Control Test (ACT) category at 24 and 52 weeks from SLS asthma. Direct costs (pharmacological and per ACT category) were estimated from Spanish public sources and literature (€, 2018). Base case analysis assumed an increased use of FF/VI from 10 to 20% within 1 year. One-way sensitivity analyses were performed.

**Results:**

Within the 775,900 asthmatic patients analysed, substitution of UC with FF/VI was associated with reduced costs due to ACT improvement, leading to potential total annual savings of €4,927,672. Sensitivity analyses ranged from €6,012,975 to €14,783,015 cost savings associated with FF/VI. An analysis considering patients only on ICS/LABA showed potential cost savings of €8,207,448.

**Conclusions:**

The improved asthma control for FF/VI compared with UC observed in SLS asthma could be translated into potential savings for the Spanish NHS. These results may be useful for decision makers.

## Introduction

Asthma is a common chronic inflammatory disorder of the airways that causes recurrent episodes of breathlessness, chest tightness and coughing [1]. It is one of the most common respiratory conditions in Europe and the prevalence is increasing. Results of the European Community Respiratory Health Survey showed large geographical differences in the prevalence of asthma [2]. The estimated prevalence of asthma in Spain is 4.90% [3] and is associated with a significant economic burden. It generates high annual costs to the Spanish National Health System (NHS). In Spain in 2016, a total of 23,125 hospital discharges and a total of 134,640 stays were caused by asthma [4]. The total annual costs of asthma are estimated to be €3022 million for patients with asthma diagnosis and the average total direct annual costs of an asthmatic patient have been estimated to reach €1533 [5]. Indirect costs associated with asthma are also considerable and are mainly due to loss of productivity resulting from absenteeism (lost work and school days) and presenteeism (self-reported impairment at work) [6]. Therefore, the availability and use of healthcare interventions that may contribute to reduce not only the clinical but also the economic burden of the disease seem crucial.

The main goal of asthma management is to achieve and maintain symptom control and minimise the future risk of exacerbations [1]. Despite the availability of effective therapies, several studies show that more than half of the asthmatic patients suffer from suboptimal control [6, 7] and results of the European REALISE study observed that only 20.1% of patients had controlled asthma [8]. Poorly controlled asthma is also associated with a reduction of patients’ health-related quality of life and with substantial healthcare resource use [9].

Nowadays, both international and national guidelines for asthma management [1, 10] are mainly based on efficacy randomised controlled trials with usually restrictive patient inclusion/exclusion criteria and this makes it difficult to extrapolate the results to everyday clinical practice. Therefore, there is a need for randomised controlled trials that are closer to usual clinical practice to assist clinicians and healthcare providers in their asthma-related decision-making processes.

The Salford Lung Study (SLS) in asthma (NCT01706198) is a phase III, multicentre, open-label, randomised controlled trial. The primary objective of which was to compare the effectiveness of initiating treatment with the once-daily combination of fluticasone furoate and vilanterol [FF/VI, 184/22 mcg and 92/22 mcg, GlaxoSmithKline, Brentford, London, United Kingdom (UK)] with usual asthma maintenance therapy over a 52-week period on asthma control and safety. The study was conducted in 74 primary care centres in Salford and South Manchester, UK, between 2012 and 2016, in a population intended to be representative of everyday clinical practice. Patients had a general practitioner’s diagnosis of symptomatic asthma (without need for spirometry) and were managed by their own primary care team. In this line, practitioners chose the appropriate therapy according to their clinical opinion within the usual care (UC) arm and subjects were randomly allocated to FF/VI or UC and thereby treatments were dispensed by community pharmacies in the usual way [11].

Results of this study showed that significantly more patients initiating treatment with FF/VI were considered responders compared to patients continuing with UC. In the FF/VI group, 71% patients achieved a relevant response vs 56% in the usual care group (%; OR 2.00; 95% CI 1.70, 2.34; *p* < 0.001), in the primary effectiveness analysis (PEA) population, a subgroup of the Intention-to-treat (ITT) population who had an Asthma Control Test (ACT) score less than 20. Response was defined as patients who achieved at week 24 an ACT total score of 20 or greater (indicating well-controlled asthma) or an increase of 3 points (the minimal clinically important difference for ACT) in ACT total score from baseline. The difference in ACT response between the FF/VI and UC groups was consistent over the 52-week period. These results were also achieved in a subset patient population for whom a combination of inhaled corticosteroids with a long-acting β2-agonist (ICS/LABA) was intended as UC [11].

To maximize the benefits of healthcare spending, economic evaluation is becoming increasingly relevant in health care decision making for resource allocation. Studies addressing the economic consequences of asthma maintenance treatments are needed to inform decision-making processes. In this sense, the objective of this analysis is to estimate the economic consequences of substitution of UC by FF/VI on the Spanish healthcare budget when applying the SLS asthma results in an everyday clinical practice Spanish population.

## Materials and methods

A cost–consequence economic model was developed in Excel and populated with the SLS Asthma results and Spanish data. A 1-year time horizon was selected, in alignment with the length of the SLS Asthma study duration, and the analysis was performed from the Spanish National Healthcare System (NHS) perspective. The model estimated the economic consequences of the current scenario, and these results were compared to a hypothetical alternative scenario in which the market shares or uses of FF/VI and UC had changed.

The analysis was performed following the recommendations of international and Spanish guidelines for economic evaluation [12–14]. All assumptions made for the analysis were validated with a panel of three clinical experts, the authors of this manuscript.

### Study comparators

Treatment comparators were chosen according to the ones evaluated in the SLS Asthma study:

- *FF/VI* Including both presentations, 92/22 mcg and 184/22 mcg.
- *UC* Defined as the maintenance treatment of asthma according to the everyday clinical practice. Patients could receive either an ICS alone or in combination with LABA.

### Model structure

**Fig. 1:**
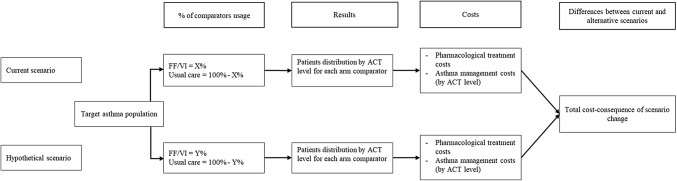
Economic model structure. *FF/VI* fluticasone furoate/vilanterol, *ACT* asthma control test

The model estimated the monetary consequences of substitution of UC by FF/VI on the Spanish health care budget when applying the SLS asthma results to the Spanish population. The model structure is shown in Fig. 1. Results were obtained in terms of pharmacological treatment costs and asthma management costs considering two scenarios:

- *Current scenario* Represented the current usage of FF/VI 92/22 mcg and 184/22 mcg and UC as observed in the current everyday clinical practice in Spain
- *Alternative scenario* Represented a hypothetical everyday clinical practice assuming an increase in usage of FF/VI with respect to the current practice.
- Lastly, the model estimated differences in costs between both scenarios.

The reference year chosen for the analysis was 2018. For that year, the current usage of FF/VI in Spain was estimated to be 10% with respect to the rest of asthma maintenance treatments available in the market (UC = 90%). For the base case analysis, in the alternative scenario, the model assumed an increase of usage of FF/VI up to 20% (UC = 80%) to explore the economic consequences of this increase. Other values of usage in the alternative scenario were also explored as part of the deterministic sensitivity analysis to assess the potential impact on the results.

### Model inputs

#### Population

**Fig. 2:**
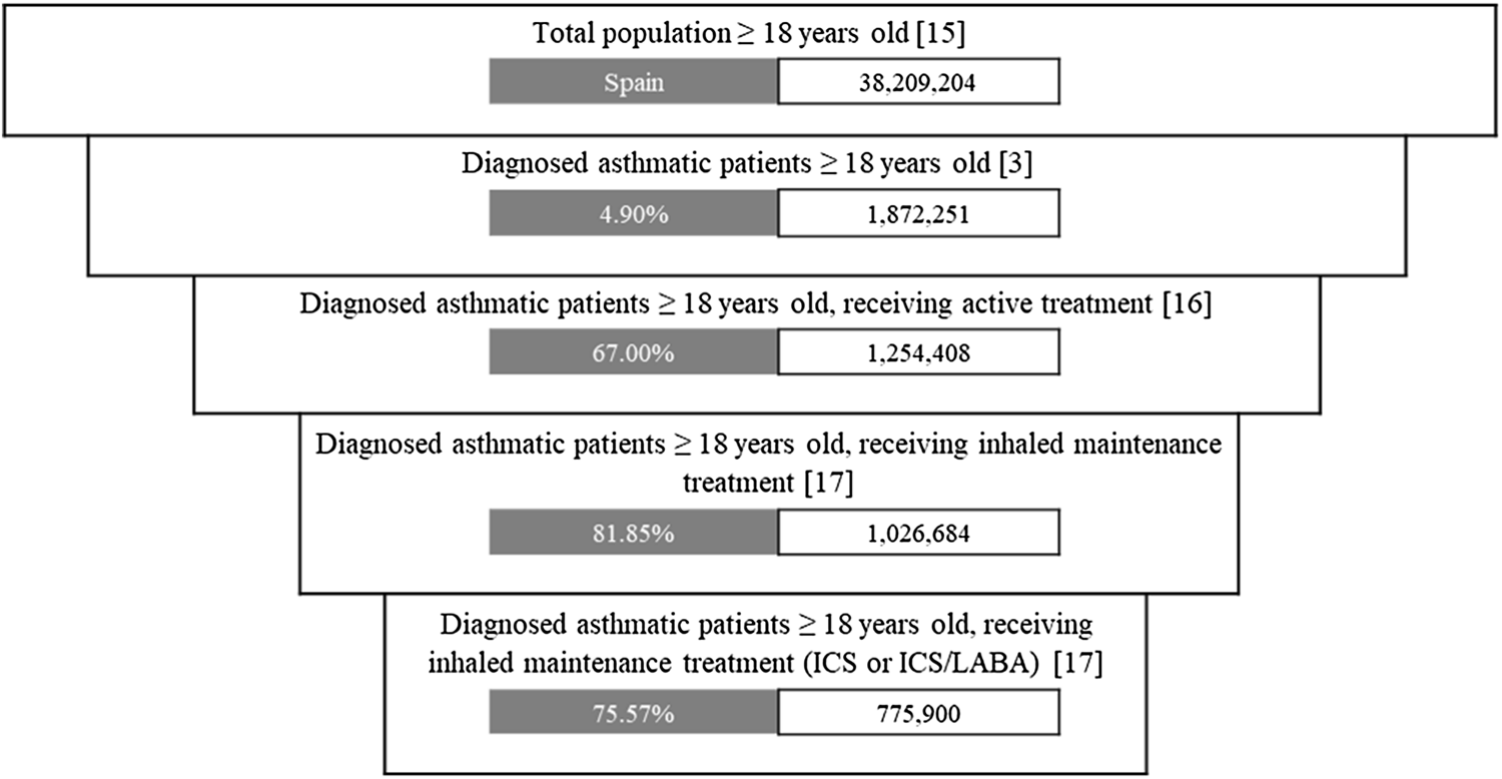
Spanish asthmatic target population estimation. *ICS* inhaled corticosteroid, *ICS/LABA* inhaled corticosteroid/long-acting beta-agonist

The Spanish population was included in alignment with the SLS asthma study inclusion criteria [11]: patients aged ≥ 18 years, with asthma diagnosis confirmation, who had a regular maintenance inhaler therapy with either ICS or ICS/LABA. Considering these criteria, the number of Spanish patients to be included in the economic model was estimated based on local data [3, 15–17]. Population estimation is detailed in Fig. 2.

#### Clinical inputs

In the SLS asthma study, primary effectiveness endpoint was defined as the percentage of patients to achieve (a) an ACT score of 20 points or greater, or (b) an increase in ACT score from baseline of three points or greater at 24 weeks (termed responders) in the PEA population [defined as the ITT patients with a baseline ACT score less than 20 (*n *= 3026)] [11]. The mean change from baseline in ACT total score at weeks 12, 24, 40 and 52 was also measured as a secondary endpoint in the ITT population; this enabled us to know the distribution of patients among each ACT category at the different time points [18].

In the present analysis, treatment effect of both comparators on asthma control was incorporated using the patient distribution among the three ACT categories (well controlled, ≥ 20; partially controlled, 16–19 and uncontrolled ≤ 15) observed during the SLS asthma study in the ITT population (*n* = 4233) [18]. It included the patient distribution at 24 weeks for the first 24 weeks of the year and at 52 weeks for the remaining 28 weeks. Results are detailed in Table 1.

**Table 1:** Model inputs for base case analysis

Variables	FF/VI 184/22 mcg and 92/22 mcg	UC group
Monthly drug costs [17, 21]	€51.52^a^	ICS = €18.75^b^ ICS/LABA = €48.25^b^
Annual drug costs (€, 2018)	€627.26	€557.31
Asthma management direct annual costs (€, 2018)^c^ [19, 20]		
Uncontrolled (ACT score ≤ 15)	€656.90	
Partially controlled (ACT score 16–19)	€1015.61	
Well-controlled (ACT score ≥ 20)	€656.90	
Assumed proportion of days covered, %	100	100
Assumed current uptake, %	10	90
Assumed new uptake, %	20	80
Percentage of subjects in each ACT category (ITT population, *n* = 4233) [18]		
Baseline		
Uncontrolled	41%	41%
Partially controlled	31%	31%
Well controlled	28%	28%
24 weeks		
Uncontrolled	20%	29%
Partially controlled	20%	25%
Well controlled	60%	46%
52 weeks		
Uncontrolled	21%	30%
Partially controlled	20%	26%
Well controlled	59%	44%

*FF/VI* fluticasone furoate/vilanterol,* ICS* inhaled corticosteroid,* ICS/LABA* inhaled corticosteroid/long-acting beta-agonist,* ACT* Asthma Control Test,* ITT* intention-to-treat

^a^Price to public plus VAT of FF/VI 184/22 and 92/22 mcg

^b^UC cost calculated as a weighted average drug cost of all available presentations at price to public plus VAT

^c^Costs annualised at updated to 2018

#### Cost inputs

##### Asthma management costs

Total asthma management costs were estimated based on the distribution of patients among the three ACT categories, as seen in the SLS Asthma (Table 1), multiplied by literature-derived costs per each ACT category. Costs per ACT category were obtained from a published Spanish prospective study of 3-month follow-up of asthmatic patients classified by ACT category that calculated the direct and indirect costs associated with each ACT level. Based on the results of this study, the average annualised direct costs (actualised to 2018) were: €656.90 for controlled asthma, were €1015.61 for partially controlled asthma and €1919.01 for uncontrolled asthma [19, 20]. Adverse events costs were not included as no differences in serious adverse events were observed between groups [11].

##### Pharmacological costs

The model estimated the annual pharmacological costs of each comparator, which were based on monthly treatment costs, calculated as acquisition cost per medication packs delivered at retail pharmacies and expressed as price to public plus value-added tax (PTP + VAT) from the Spanish Ministry of Health Catalogue published in April 2018 [21].

For FF/VI, the monthly cost for both presentations (184/22 and 92/22 mcg) was established as price per pack from published PTP + VAT [21].

The monthly cost for the UC arm was based on monthly costs of each therapeutic class, ICS and ICS/LABA, and considering the relative market shares for each class as observed in clinical practice in Spain.

According to a market research study performed in the year 2017, the relative market shares within UC applied in this analysis were 91.6% for ICS/LABA and 8.4%, for ICS [14].

To calculate the monthly costs for the therapeutic groups of ICS and ICS/LABA, all active ingredients, brands and presentations available in Spain for each class as of April 2018 were considered. Cost estimation was based on:

- Relative market shares of each presentation within the therapeutic class in the Spanish market, according to retail data for 2017 [14].
- PTP + VAT of all available presentations, detailed in Table 2 [17].

**Table 2:** Detail of retail prices used for the UC arm monthly cost estimation

Active ingredient	Brand name	Strength and pack size	Cost per pack PTP + VAT (€) [21]	Market shares (%) [17]
Inhaled corticosteroids (ICS)				
Fluticasone propionate	Flixotide Accuhaler^®^	500 mcg × 60 doses	€31.47	20
		100 mcg × 60 doses	€11.43	
	Flixotide^®^	250 mcg × 120 doses	€31.47	
		50 mcg × 120 doses	€11.43	
	Fluticasona Cipla^®^	125 mcg × 120 doses	€15.74	
Budesonide	Pulmicort Turbuhaler^®^	200 mcg × 100 doses	€13.16	70
		100 mcg × 200 doses	€13.16	
		400 mcg × 100 doses	€26.32	
	Budesonide Easyhaler^®^	200 mcg × 200 doses	€26.32	
		100 mcg × 200 doses	€13.16	
		400 mcg × 100 doses	€26.32	
	Budesonide Pulmictan^®^	50 mcg × 200 doses	€7.56	
		200 mcg × 100 doses	€11.11	
		200 mcg × 200 doses	€20.25	
	Miflonide^®^	200 mcg × 60 doses	€7.90	
		400 mcg × 60 doses	€15.80	
Beclometasone	Becotide^®^	50 mcg × 200 doses	€3.50	2
	Becloforte^®^	250 mcg × 200 doses	€18.98	
Mometasone furoate	Asmanex^®^	200 mcg × 60 doses	€29.07	3
		400 mcg × 60 doses	€51.14	
Ciclesonide	Alvesco^®^	160 mcg × 60 doses	€32.78	5
ICS/LABA fixed-dose combinations				
Fluticasone propionate/salmeterol	Seretide^®^	25/50 mcg × 120 doses	€41.28	31
		25/125 mcg × 120 doses	€41.28	
		25/250 mcg × 120 doses	€41.28	
	Seretide Accuhaler^®^	50/100 mcg × 60 doses	€41.28	
		50/250 mcg × 60 doses	€41.28	
		50/500 mcg × 60 doses	€41.28	
	Airflusal Forspiro^®^	50/250 mcg × 60 doses	€41.28	
		50/500 mcg × 60 doses	€41.28	
Budesonide/formoterol	Symbicort Turbuhaler^®^	80/4,5 mcg × 120 doses	€51,39	44
		160/4,5 mcg × 120 doses	€51.39	
	Symbicort Forte Turbuhaler^®^	320/9 mcg × 60 doses	€51.39	
	DuoResp Spiromax^®^	160/4.5 mcg × 120 doses	€51.39	
		320/9 mcg × 60 doses	€51.39	
Beclometasone/formoterol	Foster^®^	100/6 mcg × 120 doses	€51.52	19
		200/6 mcg × 120 doses	€51.52	
	Foster Nexthaler^®^	100/6 mcg × 120 doses	€51.52	
		200/6 mcg × 120 doses	€51.52	
Fluticasone propionate/formoterol	Flutiform^®^	50/5 mcg × 120 doses	€32.86	7
		125/5 mcg × 120 doses	€45.79	
		250/10 mcg × 120 doses	€71.81	

*PTP + VAT* price to public plus value added tax,* ICS* inhaled corticosteroid,* LABA* long-acting beta-agonists

The ICS/LABA cost was obtained excluding FF/VI from calculations.

Overall UC monthly costs were calculated using the following formula:

$${\text{UC}}\,{\text{monthly}}\,{\text{cost}} = (\% {\text{ICS}} \times [(P_{ 1} \times W_{1} ) + (P_{2} \times W_{2} ) + \cdots + (P_{n} \times W_{n} )]) + (\% {\text{ICS}}/{\text{LABA}} \times [(P_{{ 1^{\prime}}} \times W_{{1^{\prime}}} ) + (P_{{2^{\prime}}} \times W_{{2^{\prime}}} ) + \cdots + (P_{{n^{\prime}}} \times W_{{n^{\prime}}} )]) ,$$

where %ICS is the relative weight of the ICS therapeutic class within UC, *P*_1_ is the price of presentation 1 from the ICS therapeutic class, *W*_1_ is the relative weight of presentation 1 within the ICS therapeutic class, %ICS/LABA is the relative weight of the ICS/LABA therapeutic class within UC, *P*_1′_ is the price of presentation 1 from the ICS/LABA therapeutic class, *W*_1′_ is the relative weight of presentation 1 within the ICS/LABA therapeutic class.

In the base case analysis, complete patient compliance with both treatment arms was assumed for pharmacological cost calculation, meaning a proportion of days covered (PDC) with study medications of 100%. This value was modified to assess potential impact on study results as part of the deterministic sensitivity analyses that are detailed in Table 3.

**Table 3:** Deterministic sensitivity and alternative scenario analyses performed

Modified parameters	Base case value/s	Alternative value/s
Effectiveness results	ITT population (FF/VI vs ICS and ICS/LABA) [18]	ITT population (FF/VI vs ICS/LABA) [18]
Effectiveness results	ITT population (FF/VI vs ICS and ICS/LABA) [18]	PEA population (FF/VI vs ICS and ICS/LABA) [18]
Analysis perspective	Spanish National Healthcare System (only direct costs) [19, 20]	Society including indirect costs by ACT category: [19, 20] Uncontrolled: €540.71 Partially controlled: €49.92 Well controlled: €17.23€
Time horizon	1 year [11]	3 years (assuming an increase of usage of FF/VI in each year) (assumptions)
Usage of comparators in the alternative scenario	FF/VI 20% UC 80% (assumption)	(1) FF/VI = 30% UC = 70% (2) FF/VI = 40% UC = 60% (assumptions)
Proportion of days covered	100% (assumption)	(1) 80% [10] (2) 50% [22] (3) 33% [23]

Main assumptions, variables and inputs used for the base case analysis in the economic model are summarised in Table 1.

### Results reporting

Results were expressed as cost differences of both scenarios [in euros (€) for 2018] obtained with the following formula:

$${\text{Cost}} - {\text{consequence}} = ({\text{Pcosts}}_{\text{CS}} + {\text{AMcosts}}_{\text{CS}} ) - ({\text{Pcosts}}_{\text{AS}} + {\text{AMcosts}}_{\text{AS}} ) = \Delta \EUR ,$$

where Pcosts is the pharmacological costs, AMcosts is the asthma management costs, CS is the current scenario, and AS is the alternative scenario.

### Sensitivity analyses

To minimise the impact of uncertainty and to determine the robustness of the results, different scenarios from the base case were explored:

- It was observed that the use of ICS/LABA as maintenance treatment for asthmatic patients is widespread in Spain. To analyse the potential impact on the results of the ICS/LABA use, the effectiveness results of the ICS/LABA subgroup of the ITT population were applied as clinical inputs.
- In the SLS asthma, data were collected for the PEA population (*n* = 3026), defined as all ITT included subjects with an ACT score of less than 20 points at baseline. Therefore, an additional scenario was analysed using the patient’s distribution among the ACT categories observed in the PEA population to investigate the impact in asthma management costs compared with the base case results.
- Another alternative scenario analysis was performed from the society perspective, including loss of productivity costs by ACT level, extracted from the published literature. Annualised asthma management indirect costs and actualised to 2018 were €17.23 for well-controlled asthma, €49.92 for partially controlled and €540.71 for uncontrolled asthma [19, 20].
- To explore the results in a longer term, a 3-year time horizon was selected, where year 1 base case scenario was compared with a 20% of FF/VI use in the alternative scenario. For the 2nd year, base case was compared with a 25% use of FF/VI and for the 3rd year vs a 30% use. Differential treatment effects between comparators from the SLS Asthma study were maintained during the whole time horizon. Following Spanish guidelines recommendations, a 3% discount rate was applied to years 2 and 3 [14].

In addition, several one-way deterministic sensitivity analyses were performed by individually modifying selected parameters:

- Percentage of usage of FF/VI in the alternative scenario: in the base case, 10% of FF/VI usage was compared to 20% usage in the hypothetical scenario. To explore the economic consequences of a higher increase in usage, the same usage of FF/VI in the base case for the current scenario was compared with an increase of usage of 30% and 40% in the alternative scenarios.
- Patient compliance to drug treatments: in an everyday clinical practice, asthmatic patients are not thought to be fully compliant with their medication. Therefore, PDC was modified to 80% of days covered (average compliance treatment observed in the SLS asthma study), and 50% and 33% (based on literature [22, 23]) to analyse the impact of different compliance rates on the results.

All value modifications for the sensitivity and other scenario analyses performed are detailed in Table 3.

## Results

### Base case analysis results

A total of 775,900 asthmatic patients were estimated and included in the model, with the specified calculation methods (Fig. 2). For the base case analysis, an increase of usage of FF/VI in the alternative scenario was associated with:

- Increased drug treatment costs: accounting for an increment of €5,427,249.
- Decreased asthma management costs: accounting for €10,354,921 cost savings.

As a result, a substitution of UC with FF/VI up to 20% could lead to potential total annual cost savings of €4,927,672 for the Spanish NHS. Cost savings were mainly derived from the ACT improvement when treated with FF/VI compared with continuing with usual care.

### Deterministic sensitivity and scenario analyses results

**Fig. 3:**
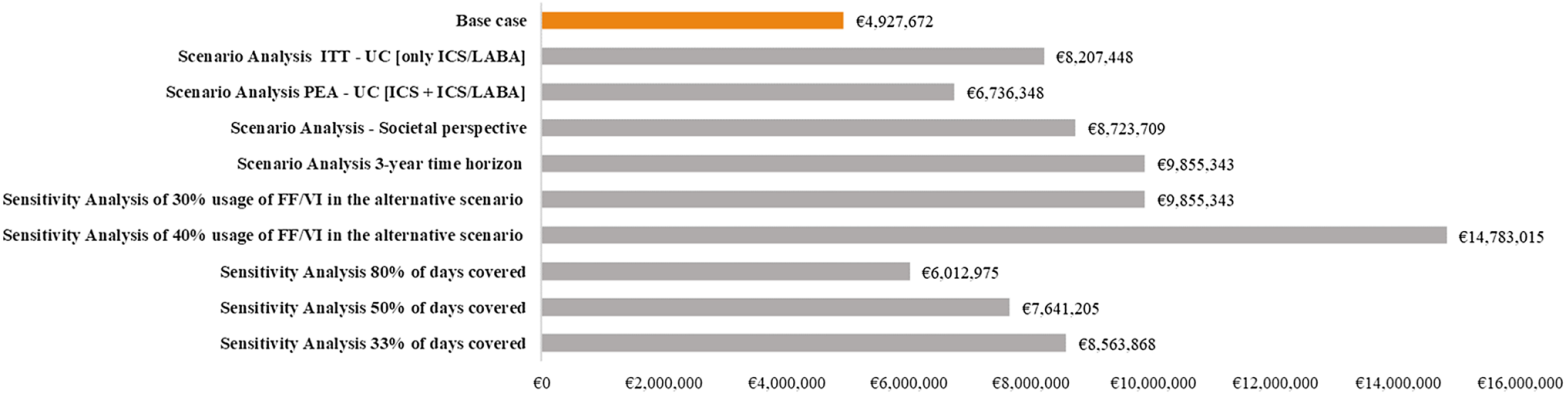
Potential cost savings of base case, sensitivity and scenario analyses. *ITT* intention-to-treat, *UC* usual care, *PEA* primary effectiveness analysis, *ICS* inhaled corticosteroid, *ICS/LABA* inhaled corticosteroid/long-acting beta-agonist

Results from all the different analyses, both deterministic and scenario analyses, were shown to be consistent with the base case, demonstrating the robustness of the base case analysis results. Cost savings ranged from €6,012,975 to €14,783,015 and are detailed in Fig. 3.

As for the deterministic sensitivity analyses, the modification of the parameter “increase of usage of FF/VI in the alternative scenario” had the highest impact on the results with respect to base case. Prolonging the time horizon to 3 years and varying the rates of use of FF/VI each year caused the highest variability of the results but remained consistent with the base case.

The scenario analysis considering patients on ICS/LABA only showed that potential cost savings increased up to €8,207,448. In addition, a similar increase in cost savings was observed, when performing the analysis from a societal perspective that included indirect costs due to loss of productivity, with cost savings of €8,723,709.

Base case and sensitivity analyses results are presented in Table 4.

**Table 4:** Economic model results

Assessed scenario	Total costs current scenario	Total costs hypothetical scenario	Potential cost savings obtained with FF/VI
Base case	€1,297,515,968	€1,292,588,296	− €4,927,672
ITT population—UC (only ICS/LABA)	€1,361,896,401	€1,353,688,953	− €8,207,448
PEA population—UC (ICS and ICS/LABA)	€1,380,900,012	€1,374,163,664	− €6,736,348
Societal perspective	€1,433,542,596	€1,424,818,887	− €8,723,709
3-year time horizon	€3,768,522,100	€3,758,879,953	− €9,642,147
FF/VI usage up to 30% in the alternative scenario	€1,297,515,968	€1,287,660,625	− €9,855,343
FF/VI usage up to 40% in the alternative scenario	€1,297,515,968	€1,282,732,953	− €14,783,015
PDC of 80%	€1,209,946,109	€1,203,933,134	− €6,012,975
PDC of 60%	€1,078,593,799	€1,070,952,594	− €7,641,205
PDC of 33%	€1,004,160,823	€995,596,955	− €8,563,868

## Discussion

The present study revealed that UC with FF/VI in Spanish asthmatic patients could lead to potential total annual savings of €4,927,672. When the effectiveness results of the ICS/LABA ITT subgroup were used as clinical input data, the cost savings increased up to €8,207,448. An increase in FF/VI usage among Spanish asthmatic patients could contribute to reduce the total economic burden associated with the management of asthma in Spain.

These results confirmed that the health benefit in terms of asthma control with FF/VI compared with UC seen in the SLS Asthma study could be translated into economic benefits for the Spanish NHS. Despite the increase of drug treatment costs related to the substitution of UC by FF/VI, they are compensated by cost savings resulting from a reduction in asthma management costs. These results become particularly relevant to the Spanish current everyday clinical practice, as it is estimated that ICS/LABA fixed-dose combinations are widely used among asthmatic maintenance treatment when compared to ICS alone, with market shares of 91.6% and 8.4%, respectively. Funding medicines in a sustainable manner is a challenge for health policy in many countries due to scarce resources. Economic evaluation can be used as a tool for healthcare policy decision makers to ensure health resources are allocated efficiently, maximizing patient outcomes.

Thus far, this is the first analysis in Spain estimating the economic impact of an increase in the use of FF/VI for asthma maintenance treatment, based on effectiveness results (SLS asthma). Previous studies performed in Spain had only assessed the cost effectiveness of asthma drug treatments and were based on clinical trials [24–26]. In line with the SLS asthma, a similar analysis was performed applying the clinical data, in terms of COPD exacerbations rate reduction, from the SLS COPD study to the Spanish population. Results from this analysis showed that an increase in usage of FF/VI for maintenance treatment in COPD patients could lead to potential annual total cost savings of €353,622.98 for the Spanish NHS [27]. Up to now, both SLS Asthma and COPD studies represent the largest effectiveness studies conducted in routine clinical practice which may help to support their everyday decision-making processes.

According to guidelines, the long-term goals of disease management are achieving good control of symptoms and maintaining normal activity levels, as well as minimising future risk of exacerbations [1]. Asthma control is a key aspect of disease treatment and a good guide to a reduced risk of exacerbations [28]. Moreover, it is significantly associated with costs and health-related quality of life in Spain and it was observed that costs were higher and health-related quality of life lower, as the level of asthma control decreased [14]. In this sense, the present analysis supports the economic consequences of an improvement in asthma control due to changes in maintenance treatments.

One limitation of this analysis could be related to the distribution of treatments in the UC arm used for cost calculations. Data from the SLS asthma show that the relative weights of 36% for ICS and 64% for ICS/LABA at the start of the study differ from the market shares observed in the current clinical everyday practice in Spain, 8.4% and 91.6%, respectively. These differences could be related to the study development time period, as the SLS asthma study was conducted between 2012 and 2016, and treatment usage trends could have varied between that time and 2018, the reference year for the present analysis. Therefore, to be more representative of the current clinical everyday practice in Spain, it was decided not to use the distribution observed in SLS Asthma.

Additionally, limitations of the data source used to obtain the asthma management costs should be considered. Costs by ACT level were obtained from a 3-month follow-up study conducted in 2013 in Spain and were then annualised and updated with Consumer Price Index (CPI) to 2018 [19, 20] following the recommendations of the guidelines for economic evaluation [12–14]. Nevertheless, up to date, no other sources to obtain asthma management costs by ACT level in Spain are available to be used as an input for the present analysis. Related to this cost data, it should also be mentioned that this observational study also included pharmacological costs as part of the asthma management cost calculation, so it may result in double counting as in the present analysis pharmacological cost has been obtained for both study arms. However, this double counting equally affects both study arms and all scenarios so minimal impact is expected, as the objective of our analysis is to explore if there are differences in asthma control costs between being treated with FF/VI or continuing their usual care treatment.

The patient population of the Salford Lung study had a general practitioner’s diagnosis of symptomatic asthma (without the need for spirometry). Although, in Salford, primary care is supported by trained physicians in respiratory, the level of misdiagnosis was not recorded, and it will certainly occur, which could lead to a disease management cost overestimation. Nevertheless, this would equally affect both study arms and both scenarios, as in the previous limitation.

Another limitation that should be mentioned could be related to the adherence to treatments. Good adherence is associated with better asthma control [29]. Due to the lack of local studies about the possible impact of adherence in effectiveness results, the different rates of days covered were only applied to pharmacological costs, both in the base case and the deterministic analyses, where the value of this parameter varied.

In addition, data from a retrospective study showed rates of compliance with asthma treatment of 33% [23]. In the present analysis for the base case, to estimate the annual costs of the pharmacological treatment, monthly costs were considered assuming that the patient would be totally compliant with their medication during the whole year, meaning a proportion of days covered of 100. Given that poor adherence to treatment is widespread in asthma, this analysis could be overestimating the true medication-related costs. Therefore, to mitigate the uncertainty of the possible impact of adherence in the results, different values for the proportion of days covered were used as part of the deterministic sensitivity analyses. Lack of adherence to ICS treatments in asthma has been not only associated with poorer clinical outcomes but also with an increase in healthcare resource and associated costs [30].

Despite the above-mentioned limitations, and in addition to the demonstrated clinical effectiveness from the SLS Asthma study, results from this analysis may be useful for evaluators and decision makers in the selection of asthma maintenance treatments in Spain.

## Conclusion

Results from the present study suggest that the improvement in asthma control with FF/VI compared with usual care, including other ICS/LABAs, as maintenance treatment of asthmatic patients in an everyday clinical setting could result in potential cost savings to the Spanish NHS, reducing the associated burden of the disease. In this sense, these results could be considered useful for decision-making processes related to asthma maintenance treatment.
